# Mutational screening of affected cardiac tissues and peripheral blood cells identified novel somatic mutations in *GATA4* in patients with ventricular septal defect^☆^

**DOI:** 10.1016/S1674-8301(11)60056-0

**Published:** 2011-11

**Authors:** Chunyan Cheng, Yuan Lin, Fan Yang, Wenjing Wang, Chong Wu, Jingli Qin, Xiuqin Shao, Lei Zhou

**Affiliations:** a Department of Cardiology, the First Affiliated Hospital of Nanjing Medical University, Nanjing, Jiangsu 210029, China;; b Department of Human Population Genetics, Institute of Molecular Medicine, Peking University, Beijing 100871, China.

**Keywords:** *GATA4*, ventricular septal defect, somatic mutation

## Abstract

The aim of this study was to examine how somatic mutations of the *GATA4* gene contributed to the genesis of ventricular septal defect (VSD). The coding and intron-exon boundary regions of *GATA4* were sequenced of DNA samples from peripheral blood cells and cardiac tissues of twenty surgically treated probands with VSD. Seven novel heterozygous variants were detected in cardiac tissues from VSD patients, but they were not detected in the peripheral blood cells of VSD patients or in 500 healthy control samples. We replicated 14 single nucleotide polymorphisms (SNPs) reported in NCBI. Bioinformatics analysis was performed to analyze the possible mechanism by which mutations were linked to VSD. Among those variants, c. 1004C>A (p.S335X) occurred in the highly conserved domain of GATA4 and generated a termination codon, which led to the production of truncated GATA4. The seven novel heterozygous *GATA4* mutations were only identified in cardiac tissues with VSD, suggesting that they are of somatic origin. A higher mutation rate in cardiac tissues than in peripheral blood cells implies that the genetic contribution to VSD may have been underestimated.

## INTRODUCTION

Ventricular septal defect (VSD) is the most common cardiac developmental defect and accounts for about 20% of the overall congenital heart diseases (CHDs). Although 40%-50% of VSDs may not present clinical symptoms and 15%-20% exhibit spontaneous closure before adolescence, 20%-30% remain patent during the patient's life time. The latter could lead to severe cardiac dysfunction and potential complications, such as infective endocarditis, aortic regurgitation, as well as symptomatic arrhythmias and atrial fibrillation[1]–[3].

Cardiac development is a complicated process that is controlled temporally and spatially, and involves an array of genes that are switched on-or-off in an orderly fashion[4]–[9]. The process is also profoundly impacted by environmental factors. Over the past few years, a great effort has been made to identify genes that are critical for cardiac development by using genetic approaches. This has resulted in the identification of a number of genes, such as *GATA4, NKX2.5, TBX5, TBX1, Smad4, FOG2,* and *MEF2*[5],[10]–[13]. Among these genes, NKX2.5 and TBX5 were found to cause atrial septum defect (ASD), while GATA4 is mainly responsible for VSD, when mutated[14]–[16].

To date, approximately 30 *GATA4* mutations have been identified since the first mutation of *GATA4* was reported, in 2003, to be a causative mutation for VSD[17]–[22]. Recently, new evidence has emerged, which showed that numerous genetic mutations are likely to be somatic, because most CHDs are sporadic and not inherited from the parents. Only a very small percentage of the disease show family aggregation. This indicates that we have possibly underestimated the genetic contribution to CHDs, due to the fact that most of the mutational screenings were carried out only on peripheral blood cells, and not on the affected cardiac tissues.

In this study, we attempted to identify whether more genetic variants of *GATA4* exist in affected cardiac tissue than in peripheral blood cells. This was accomplished by performing a mutational analysis of genomic DNA from cardiac tissues and white blood cells obtained from patients with VSD. We identified seven novel genetic variants in cardiac tissues that were not found in peripheral blood cells of VSD patients or in 500 healthy control samples. Our data suggests that the genetic contribution to somatic mutation of *GATA4* has been underestimated in sporadic VSD.

## PATIENTS AND METHODS

### Patients

Twenty patients with sporadic VSD were recruited and surgically treated in the Department of Thoracic-Cardiac Surgery of the First Affiliated Hospital of Nanjing Medical University from 2000 to 2009. Signed informed consent forms were obtained from the patients or their tegal sunogates. Septial tissues were obtained from surgically abandoned cardiac tissues. Three milliliters of peripheral blood was collected into an EDTA-anticoagulant treated tube from each participant prior to surgery. Ten septial tissues were gathered from unmatched transplant hearts that did not present VSD. All tissues were stored in liquid nitrogen until used. In addition, 500 healthy individuals from our community hospitals donated blood samples that were used as controls in this study. All participants were from Han Chinese nationality. The study procedures were approved by the Institutional Research Ethics Committee of the First Affiliated Hospital of Nanjing Medical University, and all patients signed the informed consent.

### Methods

#### Extraction of genomic DNA

Genomic DNA was extracted from freshly frozen cardiac tissues and peripheral blood leukocytes by proteinase K methods as previously described[23].

#### Primer design and DNA amplification

Seventeen pairs of primers were selected to amplify all exons and intron-exon joint regions of the *GATA4* gene. To amplify the target DNA regions, a polymerase chain reaction (PCR) was performed in a 25 µL system (1×PCR buffer, 0.05 mmol/L dNTP, 0.2 µmol/L each primer, 5 ng template of genomic DNA, and 1U *Taq* DNA polymerase). The thermal cycles included 95°C for 3 min followed by 45-50 cycles consisting of 95°C for 30 sec, 55°C for 45 sec, and 72°C for 45 sec. PCR was terminated by a final extension at 72°C for 2 min. The PCR products were then purified using PEG method and stored at 4°C prior to use.

#### DNA Sequencing

DNA sequencing was performed according to the standard protocol from the manufacturer (Applied Biosystems, Foster city, CA, USA), and 3 ng of amplified DNA fragments were used in sequencing reaction. The sequencing products were purified and subjected to sequencing using the ABI PRISM 3130XL Automatic DNA sequencer (Applied Biosystems, Foster city, CA, USA). A mutation was claimed when a variant fits the criteria of 1) missense, 2) occurrence at an evolutionarily conserved region, 3) significant change of an amino acid, and/or 4) <1% in its frequency.

#### Validation

All variants, once found in either cardiac tissues or blood cells, were tested in 10 non-VSD cardiac tissues and 500 healthy individuals. The allele frequencies were calculated.

#### Bioinformatics analysis

The potential effects of genetic variants on regulatory motif binding sites, exon-intron splicing, and miRNA binding sites were bioinformatically evaluated if they were in the 5′-UTR region, exon-intron joint regions, and 3′-UTR, respectively. The analyses were implemented using web-based tools (Human Splicing Finder at http://www.umd.be/HSF/; Searching Transcription Factor Binding Sites ver 1.3 at http://www.cbrc.jp/research/db/TFSEARCH.html; ENCODE Transcription Factor Binding Tracks at http://genome.ucsc.edu/).

## RESULTS

### Patient information

Twenty sporadic VSD patients were recruited, who were from the ages of 2.5 to 27 years (***Table 1***). Five-hundred individuals without VSD were recruited as controls for validation of mutations.

**Table 1:** Sporadic VSD patients for the detection of *GATA4* mutations

Patient ID	Age(year)	Sex
1	10	Female
2	5	Male
3	13	Female
4	6	Female
5	4	Female
6	17	Female
7	4.5	Female
8	10	Male
9	9	Male
10	3	Female
11	6	Male
12	12	Male
13	20	Female
14	19	Male
15	2.5	Male
16	6	Male
17	12	Male
18	6	Male
19	8	Female
20	27	Male

### Identification of genetic variants in *GATA4*

**Fig. 1:**
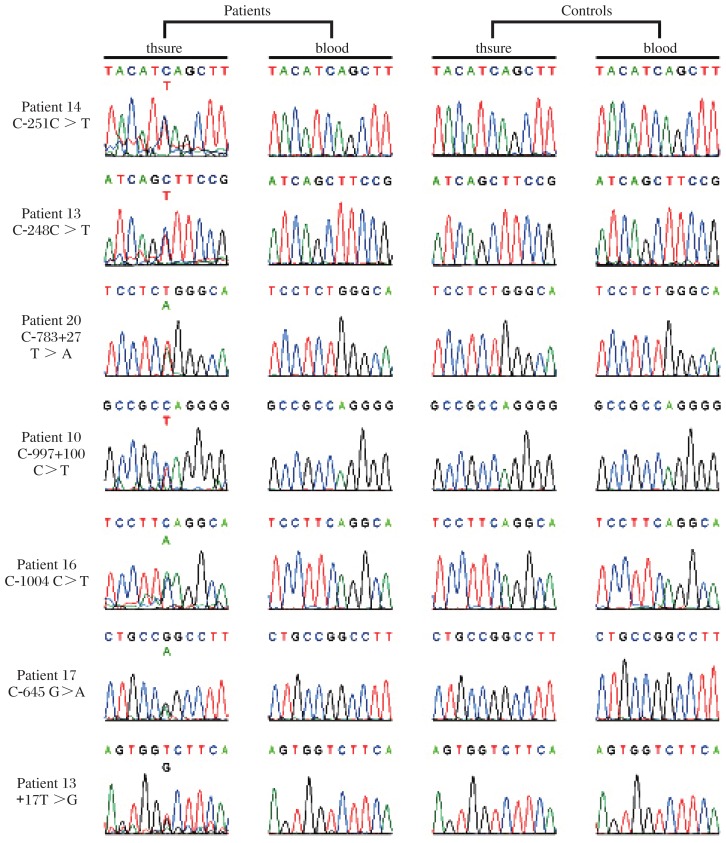
Genetic variants of *GATA4* screened in this study

Seven novel heterozygous variants were detected in the *GATA4* gene with primers listed in ***Table 2***. We designed three pairs of primers for exon2 and nine pairs of primers for exon7 respectively as the sequences of the two exons are too long. and expressed based on the international nomenclature (***Fig. 1*** and ***Table 3***). Among seven, two were in the 5′ UTR (c.-251C>T and c.-248C>T), two in introns (c.783+27T>A, intron 3; C.997+100C>T, intron 5), one in exon (c.1004C>A, exon 6), one in the 3′ UTR (c.*645G>A), and one in the 3′ flanking region (*17T>G) of the *GATA4* gene. None of these variants were detected in the control blood and tissue. We replicated fourteen reported single nucleotide polymorphisms (SNPs). No significant differences were found in the allele frequencies of these SNPs between VSD patients and normal controls.

### Functional implications

The potential functional effects of these variants were evaluated by bioinformatics analysis (***Table 3***). It was found that the c.1004C>A (p.S335X) is a nonsense mutation that leads to the production of a truncated GATA4 protein. The variant c.-248C>T shifted motif from ets-1 to CNTF, while c.783+27T>A and c.997+100C>T potentially affected RNA splicing. The variants c.*645G>A and *17T>G introduced motifs swi-5 and mef-2a, respectively.

**Table 2:** Primers for PCR and sequencing

Exon	Forward primer (5′-3′)	Reverse primer (5′-3′)
1	CTCCCTGGCGGTAGCAC	TCGGTGAAGTGAGTAGCG
2-1	CCCCGTGGCGACTTCA	ACGGCAACAACGATAAT
2-2	CTCGCCAGTCTACGTGCCCAC	GCTCCGCCGCCACTGCTGTAG
2-3	GCCGACGGAGCCGCTTACACC	CCTGCCCCGGCCCTCACG
3	TTCTCAGATGTGAGAGCTGGGCA	AAACCAGAGGATGTCCCACCAAG
4	GCCGTCACAGGTCAGA	ACAAAGGAAGAAGACAAGG
5	GAGATTGCTTAGGTGTTGC	AGGGATGTCCGATGCT
6	AAAGCCATTAGCTTGCACCCATC	GTAGCTCACTGCTTGCACCTGTG
7-1	AGTATCCACAGGGCCACCG	GGAAGATTACGCAGTGATTATGTC
7-2	CTGGGACTTGGAGGATAGC	CCCATCAGCGTGTAAAGG
7-3	GAAGCGGGTGTTGGATT	AGTCAGATTTGGTATTAGG
7-4	ACGCTGATGGGACTGGA	GATGGATGGGGCAAGGG
7-5	TGTACCTGGATGCGACG	TGTGACACGGTGAACGAA
7-6	CCCCTGGCAAAACAAGA	GCCTCCTGGACAAAAGAC
7-7	TGTCTGTCTGCTCCTCCTA	AATCTTGGTTCAAAGGTATTCTT
7-8	GGCAGAAGTCTTTTGTC	TGTGGGTTAGGGAGGGTA
7-9	CTGACTGTGGCATTACTACG	CCCCATCTAATGTCTCATGT

**Table 3:** Functional predictions of variants detected in this study

Nucleotide change	Gene region	Wt	Mt	Predicted function	Reference
c.-251C>T	5′-UTR	GCTTCCGG	GCTTCTGG	No	No
c.-248C>T	5′-UTR	GCTTCCGG	GTTTCCGG	Deletion of Ets-1	Ye *et al*.[31]
		CTTCCGGAA	TTTCCGGAA	Addition of CNTF	Wang *et al*.[32]
c.783+27T>A	Intron 3	CAGCCTCCTCTGGG	CAGCCTCCTCAGGG	Splicing activity increased by 49.02%	No
c.997+100C>T	Intron 5	CCGGGCCGCCAGGG	CCGGGCCGCTAGGG	Splicing activity decreased by 9.15%	No
c.1004C>A	Exon6	TCCTTCAGGCA	TCCTTAAGGCA	p.S335X	
c.*645G>A	3′-UTR	GCCGGC	GCCAGC	Addition of Swi5	No
*17T>G	3′-UTR	CCTTAGTGGT	CCTTAGTGGG	Addition of Mef2a	Schlesinger *et al.*[33]

## DISCUSSION

GATA4 is an important transcription factor involved in cardiogenesis and in the regulation of expression of a set of cardiac genes[10],[19],[24]–[26]. The most important finding in this study was the identification of seven novel heterozygous genetic variants within the *GATA4* gene from VSD cardiac tissue and, that the novel genes were not found within the peripheral blood cells or 500 control samples. This indicated that most of the mutations that cause VSD are likely somatic and enriched in the affected cardiac tissues. Bioinformatics analysis implied that these variants potentially affected motif-binding sites and splicing. We also identified that the variant p.S335X led to the premature termination of translation, producing a truncated GATA4 protein. No functions were predicted for c.−251C>T, suggesting it was likely a rare SNP. The variants covered whole *GATA4* from 5′ UTR, coding regions, 3′-UTR, and 3′-gene flanking region.

**Fig.2:**
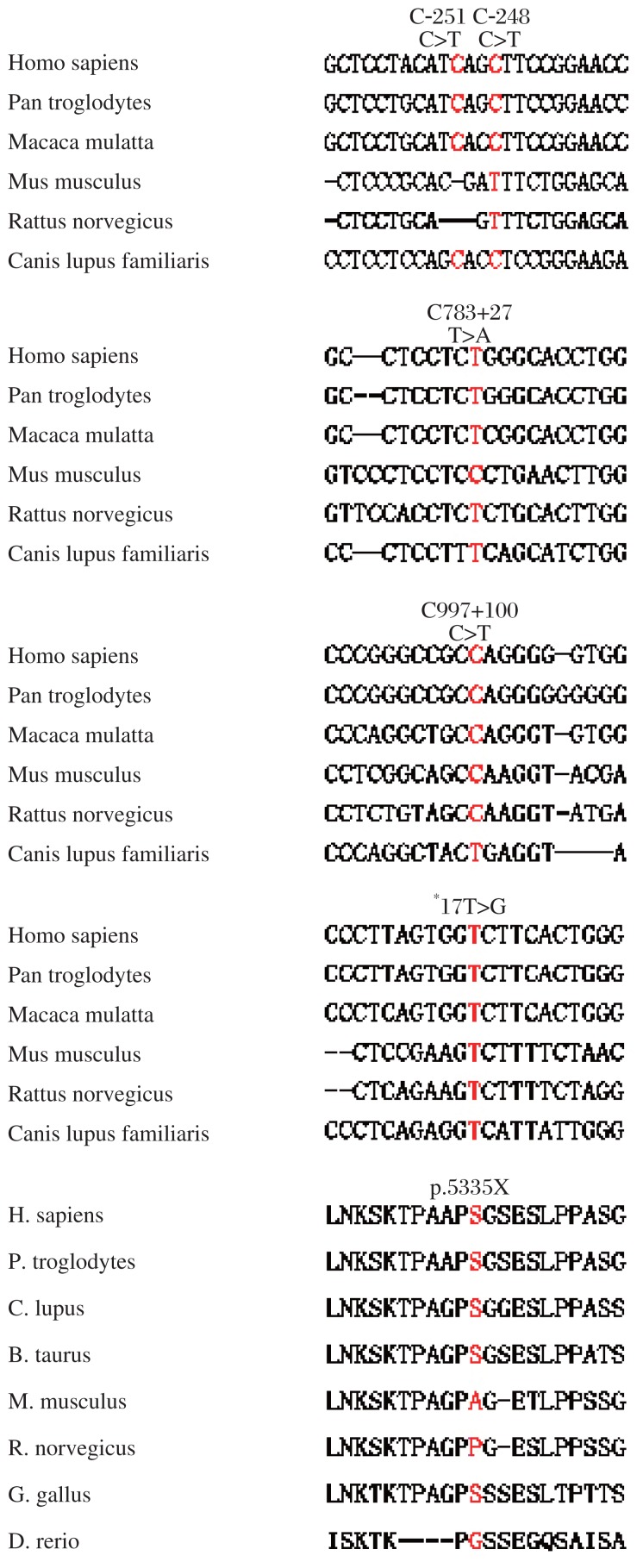
Conservative analysis of genetic variants screened in this study

Accumulating evidence has defined *GATA4* as a key gene for the pathogenesis of abnormal cardiac development. Our previous study had identified that the transcription factor *GATA4* was down-regulated in VSD patients[27]. It has been well-documented that mutations in *GATA4* are associated with a number of congenital heart defects including ventricular septal defect, atrial septal defect, Tetralogy of Fallot, endocardial cushion defect, right ventricular hypoplasia, and double-inlet left ventricle[2],[18],[25],[28],[29]. However, it has also been noticed that most congenital heart diseases are sporadic and not inherited from the parents, which suggests that genetic mutations are likely somatic. It was previously found that there is a very high mutational rate in formalin fixed malformed cardiac tissues (23 out of 68) when compared with the mutation rate in peripheral blood samples[30]. This provides a notion that we may have underestimated the genetic contribution to congenital heart disease due to the fact most of the mutational screenings were only carried out on the peripheral blood cells and not on the affected cardiac tissues. Our study, for the first time, has demonstrated that the mutational rate in freshly frozen cardiac tissue is much greater than that in peripheral blood cells (7/20 *vs* 0/20) in the Chinese population and provides direct evidence that the genetic contribution of *GATA4* to VSD is strikingly underestimated.

Genetic variants cause various functional altercations. To predict the possible mechanism by which the mutations identified in our study are associated with VSD, we performed bioinformatics analysis. We found that the mutations potentially impaired transcription and protein structure/function. For example, c.-248C>T, c.*645G>A, and *17T>G modified the regulatory motif binding sites critical for cardiac development. The variants c.783+27T>A and c.997+100C>T may potentially influence splicing. Notably, p.S335X caused a truncation of GATA4 protein, leading to a significant functional change.

In conclusion, we performed a systemic screening for genetic mutations of the *GATA4* gene in cardiac tissue and blood cells. We identified seven novel genetic variants in VSD cardiac tissues that were not found in periphery blood cells or in the 500 controls. These genetic variants may be associated with VSD through multiple mechanisms. Our data suggests that genetic contribution of somatic mutation of *GATA4* has been underestimated in sporadic VSD, and that mutational screening of genomic DNA from blood cells is insufficient for determining if a VSD patient carries a genetic mutation of *GATA4*.
